# Consensus module analysis of abdominal fat deposition across multiple broiler lines

**DOI:** 10.1186/s12864-021-07423-6

**Published:** 2021-02-10

**Authors:** Hui Yuan, Jun Lu

**Affiliations:** College of Animal Science and Technology, Northeast Agricultural University, Harbin, 150030 Heilongjiang China

**Keywords:** WGCNA, Broiler, Abdominal fat deposition, Lipid metabolism, Autophagy

## Abstract

**Background:**

Despite several RNA-Seq and microarray studies on differentially expressed genes (DEGs) between high- and low-abdominal fat deposition in different broiler lines, to our knowledge, gene coexpression analysis across multiple broiler lines has rarely been reported. Here, we constructed a consensus gene coexpression network focused on identifying consensus gene coexpression modules associated with abdominal fat deposition across multiple broiler lines using two public RNA-Seq datasets (GSE42980 and GSE49121).

**Results:**

In the consensus gene coexpression network, we identified eight consensus modules significantly correlated with abdominal fat deposition across four broiler lines using the consensus module analysis function in the weighted gene coexpression network analysis (WGCNA) package. The eight consensus modules were moderately to strongly preserved in the abdominal fat RNA-Seq dataset of another broiler line (SRP058295). Furthermore, we identified 5462 DEGs between high- and low-abdominal fat lines (FL and LL) (GSE42980) and 6904 DEGs between high- and low-growth (HG and LG) (GSE49121), including 1828 overlapping DEGs with similar expression profiles in both datasets, which were clustered into eight consensus modules. Pyruvate metabolism, fatty acid metabolism, and steroid biosynthesis were significantly enriched in the green, yellow, and medium purple 3 consensus modules. The PPAR signaling pathway and adipocytokine signaling pathway were significantly enriched in the green and purple consensus modules. Autophagy, mitophagy, and lysosome were significantly enriched in the medium purple 3 and yellow consensus modules.

**Conclusion:**

Based on lipid metabolism pathways enriched in eight consensus modules and the overexpression of numerous lipogenic genes in both FL vs. LL and HG vs. LG, we hypothesize that more fatty acids, triacylglycerols (TAGs), and cholesterol might be synthesized in broilers with high abdominal fat than in broilers with low abdominal fat. According to autophagy, mitophagy, and lysosome enrichment in eight consensus modules, we inferred that autophagy might participate in broiler abdominal fat deposition. Altogether, these studies suggest eight consensus modules associated with abdominal fat deposition in broilers. Our study also provides an idea for investigating the molecular mechanism of abdominal fat deposition across multiple broiler lines.

**Supplementary Information:**

The online version contains supplementary material available at 10.1186/s12864-021-07423-6.

## Background

Over the last few decades, broiler chickens have become a major animal protein source for the human diet [1, 2]. Production performances in commercial broilers have improved, but problems still exist, such as excessive abdominal fat deposition [3]. Excessive abdominal fat deposition not only wastes feed to producers but also increases risk to human health [4, 5]. Therefore, it is commercially relevant to investigate the molecular mechanism of abdominal fat deposition and to reduce this deposition in broilers.

At present, several studies have reported abdominal fat differentially expressed genes (DEGs) between high- and low-abdominal fat deposition in different broiler lines using RNA-Seq or microarray technology. There were 1687 DEGs identified between high- and low-abdominal fat line (FL and LL) broilers with a 2.8-fold divergence in abdominal fat percentage (AFP) at 7 weeks of age [6]. A total of 2410 DEGs were identified in abdominal fat tissue between high- and low-growth-rate (HG and LG) broilers, with a 19.2-fold divergence in AFP at 7 weeks of age [7]. A total of 286 DEGs were identified between low- and high-feed efficiency (LFE and HFE) broilers, with a 1.6-fold divergence in AFP at 7 weeks of age [8]. A total of 230 DEGs were identified between the Northeast Agricultural University (NEAU) broiler lines divergently selected for abdominal fat content with a 1.9-fold divergence in AFP at 7 weeks of age [9]. In each study, AFP was significantly different between broilers with high and low abdominal fat deposition, but the DEGs were not the same. The different DEGs among these studies may be due to the differences in lines, sample sizes, analytical methods, experimental technologies, and other factors. The differences among these studies may disturb further research on abdominal fat deposition in broilers. Consensus modules are comprised of genes tightly coexpressed in both datasets, so consensus modules exhibit a degree of preservation between the two data networks [10, 11]. Therefore, it is important to construct a consensus gene coexpression network across multiple broiler lines and to detect consensus modules correlated with abdominal fat deposition. However, to our knowledge, this work has rarely been reported in broilers.

In the current study, to explore the molecular mechanism of abdominal fat deposition across multiple broiler lines, we constructed a consensus gene coexpression network across GSE42980 (12 FL and 12 LL samples) and GSE49121 (8 HG and 8 LG samples) and detected consensus modules associated with abdominal fat deposition. Then, we tested the preservation of consensus modules in abdominal fat RNA-Seq data of another broiler line (SRP058295). The ClusterProfiler software package was used to predict Kyoto Encyclopedia of Genes and Genomes (KEGG) pathways and Gene Ontology (GO) terms associated with the genes within consensus modules. Our study provides an idea for investigating abdominal fat deposition across multiple broiler lines.

## Results

### RNA-Seq data collection

To reduce the influence of nongenetic factors, we collected public abdominal fat gene expression profile datasets of broilers under the same age, sex, and normal feed conditions. Broiler chickens were divergently selected over seven generations for either high (FL) or low (LL) abdominal fat at similar feed intake and body weight, and FL and LL broilers exhibited a 2.8-fold divergence in AFP at 7 weeks of age [6, 12]. GSE42980 is an abdominal fat RNA-Seq dataset of male broilers at 7 weeks of age that consists of 12 FL and 12 LL samples [6]. Broiler chickens were divergently selected over 30 generations for either high (HG) or low (LG) growth, and HG and LG broilers were different in AFP (19.2-fold) and body weight (3.2-fold) at 7 weeks of age [7]. GSE49121 is an abdominal fat RNA-Seq dataset of male broilers that consists of eight HG and eight LG samples. Because two pairwise broiler lines (FL and LL, HG and LG) are divergently selected over several generations and exhibit significant divergence in AFP at 7 weeks of age, the four broiler lines are good genetic models for abdominal fat deposition research. SRP058295 is an abdominal fat RNA-Seq dataset of male broilers [8]. Although broiler chickens from the SRP058295 dataset were not genetically selected, they exhibited divergence in AFP at 7 weeks of age from normal feed. In addition, all broilers in GSE42980, GSE49121, and SRP058295 were fed a normal diet. Therefore, the SRP058295 dataset was used to validate the preservation of consensus modules across two datasets. In summary, we successfully downloaded the GSE42980, GSE49121, and SRP058295 datasets for subsequent WGCNA analyses.

### QC and mapping of RNA-Seq reads

The average number of raw reads across the samples within GSE42980, GSE49121, and SRP058295 was 54.97 M (Additional file 1: Table S1). To ensure correct results in further analyses, low-quality reads were filtered, and the average number of high-quality reads was 48.97. The average percentage of high-quality reads mapped to the chicken genome was 91.65% across all samples within the three datasets (Additional file 1: Table S1). The average percentage of high-quality reads mapping to exonic regions was 80.02% (Additional file 1: Table S1).

### Hierarchical cluster analyses

In the current study, a hierarchical cluster of GSE42980 showed that 12 FL and 12 LL samples were fully separated (Additional file 2: Figure S1, a). A hierarchical cluster of GSE49121 showed that eight high- (HG) and eight low-abdominal fat deposition (LG) samples were fully separated (Additional file 2: Figure S1, b). Therefore, 24 samples within GSE42980 and 16 samples within GSE49121 were used to construct a consensus network and identify differentially expressed genes (DEGs).

### Differentially expressed gene analyses

To investigate abdominal fat gene expression differences across two pairwise comparisons, 5462 and 6904 significant DEGs were identified in FL vs. LL and HG vs. LG, respectively. There were 2846 upregulated and 2616 downregulated DEGs in the FL vs. LL comparison (Additional file 3: Table S2). Relevant genes of lipid metabolism pathways included pyruvate dehydrogenase (lipoamide) alpha 1 (*PDHA1*), dihydrolipoamide S-acetyltransferase (*DLAT*), peroxisome proliferator-activated receptor gamma (*PPARG*), retinoid X receptor gamma (*RXRG*), and acyl-CoA oxidase 1 (*ACOX1*), all of which were significantly upregulated in the FL vs. LL comparison (Additional file 3: Table S2).

There were 3324 upregulated and 3580 downregulated DEGs in the HG vs. LG comparison (Additional file 3: Table S2). Relevant genes of lipid metabolism pathways included diacylglycerol O-acyltransferase 2 (*DGAT2*), acyl-CoA synthetase long chain family member 4 (*ACSL4*), succinate-CoA ligase ADP-forming beta subunit (*SUCLA2*), 7-dehydrocholesterol reductase (*DHCR7*), and acyl-CoA synthetase bubblegum family member 2 (*ACSBG2*), all of which were significantly upregulated in the HG vs. LG comparison (Additional file 3: Table S2).

Common DEGs across two pairwise comparisons were identified according to the following criteria: common DEGs were significant DEGs (FDR ≤ 0.05) within both pairwise comparisons and had the same expression trend across two pairwise comparisons, i.e., upregulated within both comparisons, and vice versa. A total of 2020 common DEGs were shared across the two pairwise comparisons, among which many lipid metabolism genes were significantly upregulated in the FL vs. LL and HG vs. LG, such as *PDHA1*, *PPARG*, *RXRG*, *ACOX1*, *ACSL4*, *SUCLA2*, *DHCR7*, *ACSBG2*, acetyl-CoA acetyltransferase 2 (*ACAT2*), glycerol-3-phosphate acyltransferase 3 (*GPAT3*), glycerol-3-phosphate dehydrogenase 2 (*GPD2*), patatin like phospholipase domain containing 7 (*PNPLA7*), ethanolamine-phosphate phospho-lyase (*ETNPPL*), CD36 molecule (*CD36*), G protein subunit alpha i3 (*GNAI3*), fatty acid synthase (*FASN*), acyl-CoA dehydrogenase, C-2 to C-3 short chain (*ACADS*), palmitoyl-protein thioesterase 1 (*PPT1*), 3-hydroxyacyl-CoA dehydratase 1 (*HACD1*), fatty acid desaturase 2 (*FADS2*), fatty acid binding protein 3 (*FABP3*), stearoyl-CoA desaturase (*SCD*), 3-hydroxyacyl-CoA dehydratase 2 (*HACD2*), and malonyl-CoA-acyl carrier protein transacylase (*MCAT*) (Additional file 3: Table S2). The genes associated with autophagy were identified among common DEGs, such as unc-51 like autophagy activating kinase 2 (*ULK2*), autophagy related 3 (*ATG3*), autophagy related 12 (*ATG12*), autophagy related 9A (*ATG9A*), autophagy related 4B cysteine peptidase (*ATG4B*), microtubule associated protein 1 light chain 3 alpha (*MAP1 LC3A*), microtubule associated protein 1 light chain 3 beta (*MAP1 LC3B*), beclin 1 (*BECN1*), phosphate cytidylyltransferase 2, ethanolamine (*PCYT2*), and selenoprotein I (*SELENOI*) (Additional file 3: Table S2).

### Gene coexpression network construction

**Fig. 1:**
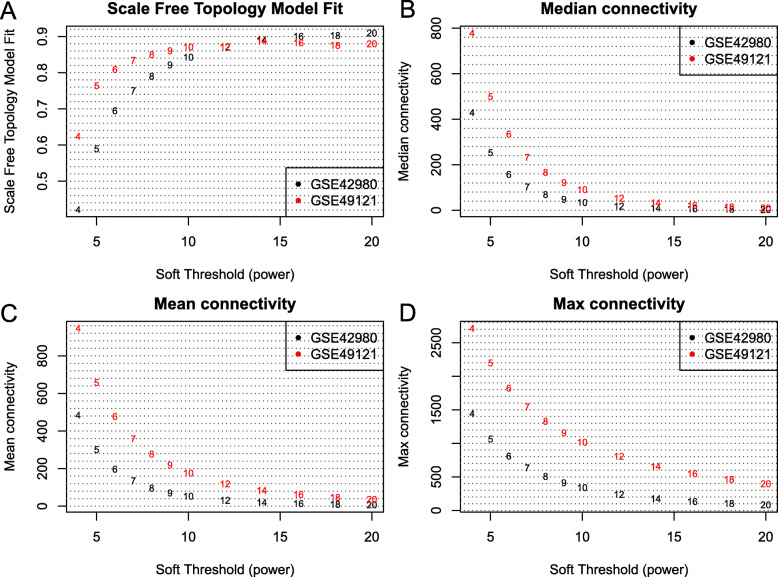
Soft threshold and network connectivity. **a** The GSE42980 and GSE49121 networks formed a scale-free network (y-axis, R^2^ > 0.85) when a soft threshold (power) was set to 14. **b**, **c**, **d** Median, mean, and max connectivities revealed node connectivity in the scale-free network when the soft threshold was set to 14

After genes with low expression were removed, a total of 13,626 genes were used to construct a consensus gene coexpression network across the two datasets (GSE42980 and GSE49121). A soft threshold (power) of 14 was selected to construct a scale-free network within the two datasets (Fig. 1). Then, we separately constructed gene coexpression networks for GSE42980 and GSE49121 with the same parameters (power = 14, minModuleSize = 30, deepSplit = 2, and mergeCutHeight = 0.3). A total of 37 and 17 gene coexpression modules were detected within GSE42980 and GSE49121, respectively (Additional file 4: Figure S2, b-c). Finally, we constructed a consensus gene coexpression network across two datasets with these parameters (power = 14, minModuleSize = 30, deepSplit = 2, and mergeCutHeight = 0.3), and a total of 45 consensus modules were identified in this consensus network (Table 1; Additional file 4: Figure S2, a).

**Table 1:** Summary of consensus modules across two datasets

Modules	Gene count	Modules	Gene count	Modules	Gene count
black	497	grey60	169	purple	295
blue	1095	ivory	36	red	815
brown	1051	lightcyan	173	royalblue	147
cyan	179	lightcyan1	44	saddlebrown	102
darkgreen	133	lightgreen	164	salmon	237
darkgrey	127	lightsteelblue1	48	sienna3	86
darkmagenta	86	lightyellow	163	skyblue	103
darkolivegreen	89	magenta	316	skyblue3	79
darkorange	117	mediumpurple3	52	steelblue	98
darkred	133	midnightblue	178	tan	246
darkturquoise	128	orange	121	turquoise	2288
floralwhite	34	orangered4	54	violet	97
green	836	paleturquoise	97	white	116
greenyellow	248	pink	408	yellow	1007
grey	977	plum1	71	yellowgreen	86

**Fig. 2:**
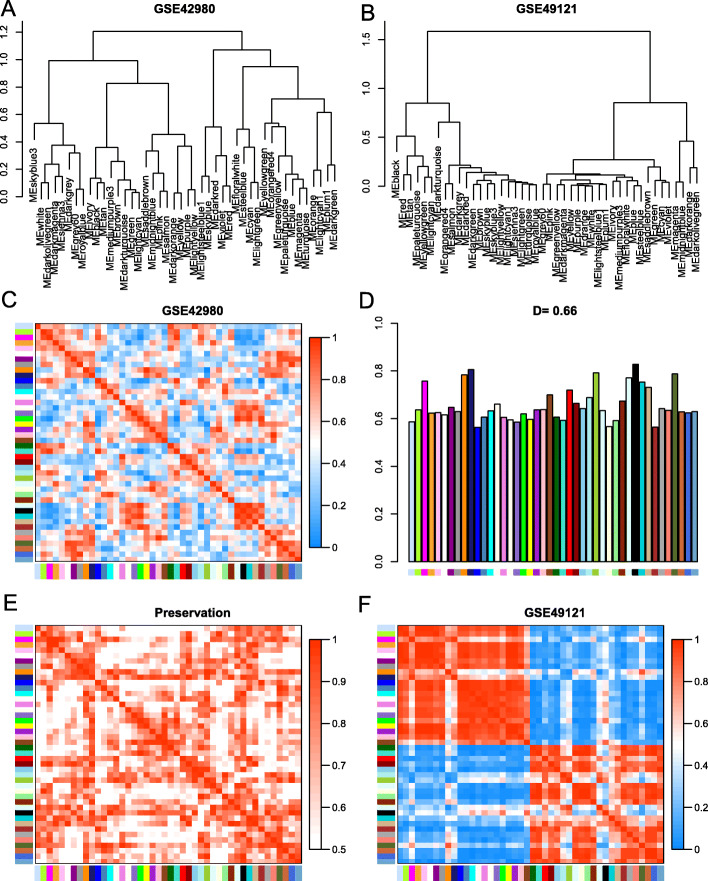
Differential eigengene network analyses across two datasets. Clustering dendrograms of consensus module eigengenes within GSE42980 (**a**) and GSE49121 (**b**). Heatmaps of consensus module eigengene adjacencies in GSE42980 (**c**) and GSE49121 (**d**). Each row and column represents one consensus module eigengene. Red indicates high adjacency (positively correlated), and green indicates low adjacency (negatively correlated) within the heatmap. Adjacency heatmap for the GSE42980 and GSE49121 preservation networks (**e**). Each row and column represents a consensus module, and the high saturation of red means high adjacency. Barplot of the preservation relationship for each consensus module eigengene between two networks (**f**). Each colored bar represents the consensus module eigengene, and bar height represents preservation of the consensus module eigengene. The density value D = 0.66 reflects overall network preservation

Clustering dendrograms of GSE42980 and GSE49121 revealed a degree of preservation of consensus modules across the two datasets (Fig. 2a-b), and heatmaps showed similar results (Fig. 2 c, and f). The density value D = 0.66 reflected an overall preservation of consensus modules across the two networks (Fig. 2d). The adjacency heatmap indicated a preservation network across the two datasets, and each row and column corresponded to a consensus module (Fig. 2e).

### Consensus modules correlated with abdominal fat deposition

**Fig. 3:**
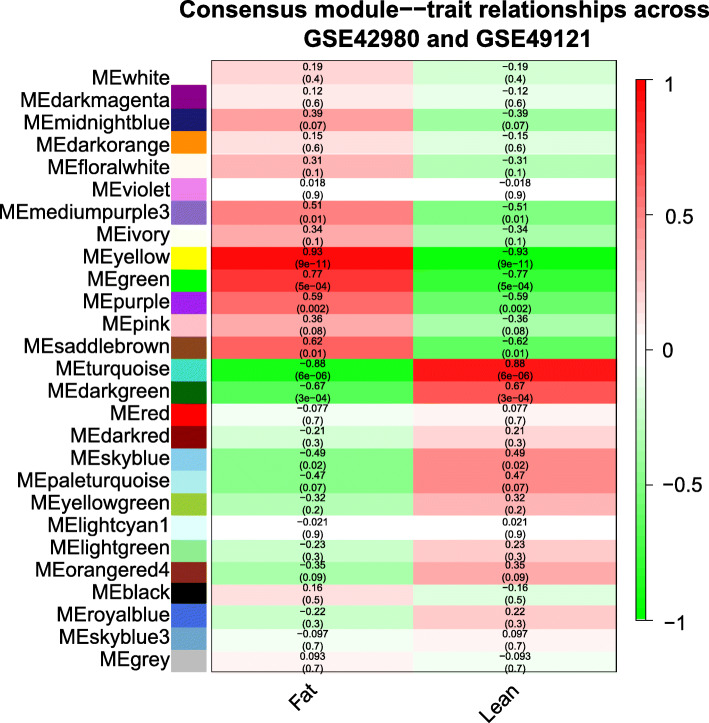
Consensus module-abdominal fat deposition relationship. Red indicates a positive correlation, and green means a negative correlation. The top number in each cell indicates the correlation coefficient, and the bottom number represents the correlation significance (*p*-value)

To detect consensus modules associated with abdominal fat deposition, we calculated the correlation between each of the consensus modules and abdominal fat deposition across the two datasets. Eight consensus modules (dark green, green, medium purple 3, purple, saddle brown, sky blue, turquoise, and yellow) were significantly correlated with abdominal fat deposition across the two datasets, and the correlation of the yellow consensus module was the highest (*r* = 0.93, *p* = 9 × 10^−11^) (Fig. 3). A total of 4816 genes were identified within eight consensus modules (Table 1). There were 13 consensus modules significantly correlated with fat deposition within GSE42980, and the correlation of the blue consensus module was the highest (*r* = 0.93, *p* = 3 × 10^− 10^) (Additional file 5: Figure S3, a). A total of 27 consensus modules were significantly correlated with abdominal fat deposition within GSE49121, and the correlation of the steel blue consensus module was the highest (*r* = 0.99, *p* = 2 × 10^− 12^) (Additional file 5: Figure S3, b).

### Preservation of interested consensus modules

**Fig. 4:**
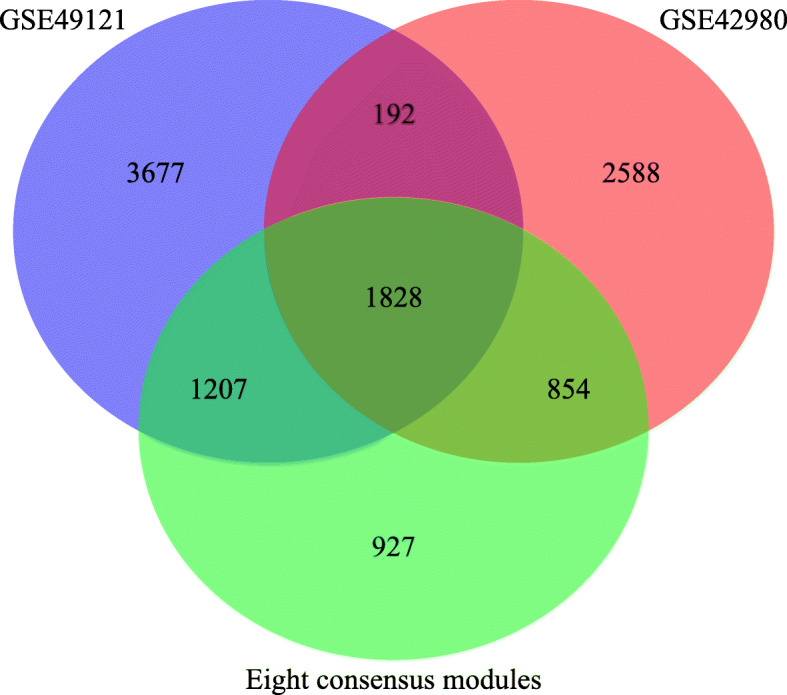
Venn diagram of genes within eight consensus modules and DEGs within GSE42980 and GSE49121. The GSE42980 circle indicates the number of DEGs within the FL vs. LL comparison; the GSE49121 circle indicates the number of DEGs within the HG vs. LG comparison; and the circle of eight consensus modules represents the number of genes within eight consensus modules

To verify the preservation of eight consensus modules across two datasets, we detected how many genes among the 2020 common DEGs were in the eight consensus modules. The results showed that a total of 1828 genes (90.5% of common DEGs) were detected in the eight consensus modules (Fig. 4).

**Fig. 5:**
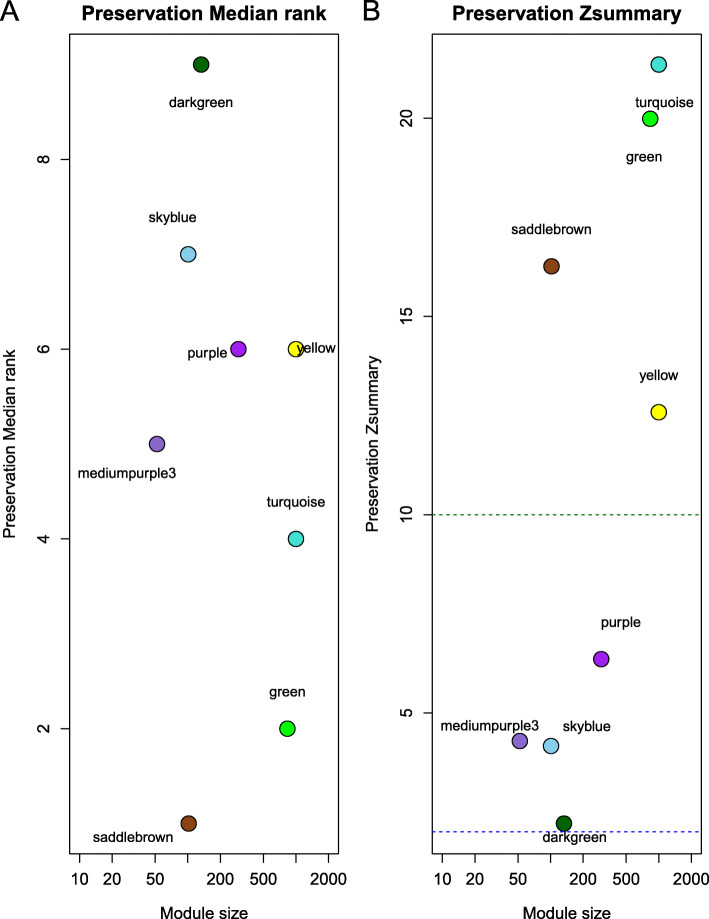
Preservation analyses of eight consensus modules. The median rank of observed preservation statistics (y-axis) of eight consensus modules was tested in the SRP058295 network as a function of module size (x-axis). Each point represents a module labeled by the text label and color (**a**). Preservation of eight consensus modules tested in the SRP058295 dataset using *Zsummary*. *Zsummary* > 10 indicates strong evidence of preservation; 2 < *Zsummary* < 10 indicates weak to moderate evidence of preservation; *Zsummary* < 2 indicates no evidence of preservation (**b**)

To test whether the eight consensus modules were stable in other broiler lines, preservation of eight consensus modules was tested in another broiler abdominal fat RNA-Seq dataset (SRP058295). The *Zsummary* results showed that the turquoise, green, saddle brown, and yellow modules were strongly preserved (*Zsummary* > 10) and the dark green, purple, medium purple 3, and skyblue modules were moderately preserved (2 < *Zsummary* < 10) (Fig. 5).

### Functional enrichment of interest consensus modules

Based on the KEGG and GO databases, 72 pathways and 749 GO terms were significantly enriched in eight consensus modules (Table 2; Additional file 6: Table S3). Glycerolipid metabolism, glycerophospholipid metabolism, and adipocytokine signaling pathways were significantly enriched in the purple consensus module, and relevant genes of the three pathways, *GPAT3*, *GPD2*, *PNPLA7*, *ETNPPL*, and *CD36,* were identified. Steroid biosynthesis was significantly enriched in the medium purple 3 module, and relevant genes, *DHCR7* and sterol-C5-desaturase (*SC5D*), were identified. Pyruvate metabolism, glyoxylate and dicarboxylate metabolism, and propanoate metabolism pathways were significantly enriched in the green module, and relevant genes of the three pathways, *PDHA1*, *ACOX1*, and *SUCLA2,* were identified. The PPAR signaling pathway was significantly enriched in the green consensus module, and relevant genes of this pathway, *RXRG*, *ACOX1*, and *ACSL4,* were identified. The fatty acid metabolism pathway was significantly enriched in the medium purple 3 and yellow consensus modules, and relevant genes of this pathway, *ACSBG2*, *ACAT2*, *FASN*, *ACADS*, *PPT1*, *HACD1*, *FADS2*, *HACD2*, and *MCAT,* were identified. Autophagy, lysosome, and mitophagy pathways were significantly enriched in the medium purple 3 and yellow consensus modules, and the relevant genes of the three pathways, *ULK2*, *BECN1*, *MAP1 LC3A*, *MAP1 LC3B*, *ATG3*, *ATG4B*, *ATG12*, and *ATG9A,* were identified in the consensus modules (Additional file 6: Table S3).

**Table 2:** KEGG enrichment in eight consensus modules

Consensus modules	Pathway ID	Description	*p*-value	Gene count
darkgreen	gga04010	MAPK signaling pathway	0.017564	6
	gga03420	Nucleotide excision repair	0.037826	2
	gga03440	Homologous recombination	0.037826	2
	gga00514	Other types of O-glycan biosynthesis	0.04737	2
green	gga00190	Oxidative phosphorylation	1.8E-06	22
	gga01200	Carbon metabolism	4.44E-05	18
	gga03050	Proteasome	4.45E-05	10
	gga00020	Citrate cycle (TCA cycle)	0.000212	8
	gga00480	Glutathione metabolism	0.000587	10
	gga04216	Ferroptosis	0.000891	8
	gga04144	Endocytosis	0.000966	27
	gga00970	Aminoacyl-tRNA biosynthesis	0.001063	9
	gga04141	Protein processing in endoplasmic reticulum	0.00317	19
	gga04146	Peroxisome	0.003185	12
	gga04130	SNARE interactions in vesicular transport	0.007734	6
	gga00563	Glycosylphosphatidylinositol (GPI)-anchor biosynthesis	0.009851	5
	gga00630	Glyoxylate and dicarboxylate metabolism	0.010798	6
	gga00620	Pyruvate metabolism	0.012614	6
	gga03020	RNA polymerase	0.014359	5
	gga00982	Drug metabolism - cytochrome P450	0.019328	6
	gga00532	Glycosaminoglycan biosynthesis - chondroitin sulfate / dermatan sulfate	0.027023	4
	gga00980	Metabolism of xenobiotics by cytochrome P450	0.028142	6
	gga03320	PPAR signaling pathway	0.03988	8
	gga01210	2-Oxocarboxylic acid metabolism	0.042742	3
	gga00640	Propanoate metabolism	0.045424	5
mediumpurple3	gga00100	Steroid biosynthesis	2.94E-12	7
	gga00900	Terpenoid backbone biosynthesis	4.86E-08	5
	gga04140	Autophagy - animal	0.005787	4
	gga04136	Autophagy - other	0.011041	2
	gga00071	Fatty acid degradation	0.01515	2
	gga01212	Fatty acid metabolism	0.036839	2
	gga04137	Mitophagy - animal	0.047564	2
purple	gga00534	Glycosaminoglycan biosynthesis - heparan sulfate / heparin	0.00728	3
	gga00561	Glycerolipid metabolism	0.018023	4
	gga00051	Fructose and mannose metabolism	0.020612	3
	gga00564	Glycerophospholipid metabolism	0.021563	5
	gga04920	Adipocytokine signaling pathway	0.022325	4
	gga00260	Glycine, serine and threonine metabolism	0.023889	3
	gga00532	Glycosaminoglycan biosynthesis - chondroitin sulfate / dermatan sulfate	0.040008	2
	gga04141	Protein processing in endoplasmic reticulum	0.046056	6
	gga00520	Amino sugar and nucleotide sugar metabolism	0.046627	3
saddlebrown	gga00190	Oxidative phosphorylation	0.001016	6
	gga04910	Insulin signaling pathway	0.001322	6
	gga04261	Adrenergic signaling in cardiomyocytes	0.008768	5
	gga04744	Phototransduction	0.022229	2
	gga00860	Porphyrin and chlorophyll metabolism	0.026023	2
	gga04270	Vascular smooth muscle contraction	0.03332	4
	gga04371	Apelin signaling pathway	0.034198	4
	gga04130	SNARE interactions in vesicular transport	0.034327	2
	gga03015	mRNA surveillance pathway	0.039714	3
	gga04120	Ubiquitin mediated proteolysis	0.04475	4
skyblue	gga05164	Influenza A	1.06E-05	8
	gga05168	Herpes simplex virus 1 infection	0.001127	6
	gga04622	RIG-I-like receptor signaling pathway	0.01011	3
	gga03440	Homologous recombination	0.036151	2
	gga04623	Cytosolic DNA-sensing pathway	0.047223	2
turquoise	gga03018	RNA degradation	0.000639	18
	gga04070	Phosphatidylinositol signaling system	0.000808	21
	gga00562	Inositol phosphate metabolism	0.01109	15
	gga04010	MAPK signaling pathway	0.011485	42
	gga04340	Hedgehog signaling pathway	0.017356	10
	gga04912	GnRH signaling pathway	0.026294	16
	gga00051	Fructose and mannose metabolism	0.039864	8
	gga00600	Sphingolipid metabolism	0.04698	10
yellow	gga04141	Protein processing in endoplasmic reticulum	0.000926	20
	gga05132	Salmonella infection	0.001751	26
	gga04142	Lysosome	0.00193	16
	gga00532	Glycosaminoglycan biosynthesis - chondroitin sulfate / dermatan sulfate	0.004474	5
	gga00510	N-Glycan biosynthesis	0.023771	7
	gga04137	Mitophagy - animal	0.028998	8
	gga01212	Fatty acid metabolism	0.038571	7
	gga04070	Phosphatidylinositol signaling system	0.040766	10

To detect gene coexpression modules associated with abdominal fat deposition, we performed the enrichment of KEGG pathways and GO terms of modules within GSE42980 and GSE49121, respectively. There were 116 pathways and 1427 GO terms significantly enriched in the genes of modules significantly correlated with abdominal fat deposition within the GSE42980 dataset (Additional file 6: Table S3). A total of 79 pathways and 849 GO terms were significantly enriched in the genes of modules significantly correlated with the GSE49121 dataset (Additional file 6: Table S3).

## Discussion

Broiler chickens have been selected to increase production performance, but these gains are also accompanied by excessive abdominal fat deposition [4]. The research results are different among different broiler lines according to published literature [6–9, 12]. Therefore, to identify the genes associated with abdominal fat deposition across multiple broiler lines, we constructed a consensus gene coexpression network and identified eight consensus modules significantly correlated with abdominal fat deposition across the GSE42980 and GSE49121 datasets. Then, the preservation of eight consensus modules was verified in another broiler abdominal fat RNA-Seq dataset (SRP058295). Finally, functional enrichment analysis associated with the genes within eight consensus modules was performed.

Adipose tissue expansion is the result of adipogenesis and lipid droplet accumulation in adipocytes, and lipid droplets are an organelle for storing fat with a hydrophobic core of triacylglycerols (TAGs) and cholesterol esters [5]. Based on the pyruvate metabolism pathway in the KEGG database, PDHA1 and DLAT convert pyruvate into acetyl-CoA, which is the ingredient for de novo synthesis of fatty acids. FASN is a key enzyme catalyzing the synthesis of fatty acids, and the FASN concentration of a tissue determines the synthesis capacity of fatty acids in this tissue by a de novo pathway [13]. The expression level of *ACSBG2* mRNA is significantly upregulated during abdominal fat-derived preadipocyte differentiation of chickens [14, 15]. The fatty acid metabolism genes *FADS2* and *SCD* are significantly upregulated in chickens with high abdominal fat content and associated with the PPAR signaling pathway [16]. In our current study, the pyruvate metabolism pathway and fatty acid metabolism pathway were significantly enriched in the green, yellow, and medium purple 3 consensus modules, and relevant genes of the two pathways, *PDHA1*, *FASN*, *ACSBG2*, *FADS2* and *SCD*, were identified. The five genes were significantly upregulated in both the FL vs. LL and HG vs. LG, and *DLAT* was significantly upregulated in the FL compared with the LL.

The PNPLA protein family plays key roles in lipid droplet homeostasis, phospholipid metabolism, and triglyceride hydrolysis [17]. PNPLA7 regulates very low density lipoprotein (VLDL) secretion by modulating apolipoprotein E (ApoE) stability and is positively correlated with plasma TAG levels in mice [18]. The expression of *GPAT3* is significantly upregulated during adipocyte differentiation in mice, and overexpression of *GPAT3* results in increased TAG in mammalian cells [19]. As the predominant enzyme for TAG storage, DGAT2 catalyzes and accounts for nearly TAG synthesis [20]. DHCR7 and SC5D are pivotal enzymes for catalyzing cholesterol synthesis, and two impaired enzymes lead to a deficiency of cholesterol synthesis [21]. In the current study, glycerolipid metabolism, glycerophospholipid metabolism, and steroid biosynthesis pathways were significantly enriched in purple and medium purple 3 consensus modules, and *PNPLA7*, *GPAT3*, *DHCR7*, *DGAT2*, and *SC5D* were identified in the three lipid metabolism pathways. *PNPLA7*, *GPAT3*, and *DHCR7* were significantly upregulated in both the FL vs. LL and HG vs. LG, and *DGAT2* and *SC5D* were significantly upregulated in the HG compared with the LG lines. Therefore, we inferred that the synthesis of fatty acids, TAG, and cholesterol in broilers with high abdominal fat deposition was greater than that in broilers with low abdominal fat deposition.

Abdominal fat deposition is a complex biological process that is controlled by a series of critical genes in signaling pathways. In chickens, *PPARG* is a well-known critical gene that is reported to positively regulate adipogenesis, lipid metabolism, adipocyte differentiation, and abdominal fat deposition [22–24]. RXRG positively regulates the PPAR signaling pathway and promotes the expression of *FABP3* and *SCD* [25]. Active immunization with CD36 results in decreased visceral fat of male broilers [26]. In the current study, the PPAR and adipocytokine signaling pathways were significantly enriched in the green and purple consensus modules, respectively, and relevant genes of the two pathways, *PPARG*, *RXRG*, *FABP3*, *SCD*, and *CD36*, were identified and significantly upregulated in both the FL vs. LL and HG vs. LG.

Autophagy is a process in which cytoplasmic macromolecules and organelles are degraded by lysosomes [27] and plays a crucial role in adipose tissue homoeostasis [28]. The biological functions of autophagy in adipose tissue have been studied in humans and model animals, but autophagy studies have rarely been reported in chicken abdominal fat. The autophagy process consists of six main steps: phagophore initiation, nucleation, and expansion; autophagosome formation, autophagosome-lysosome fusion, and lysosomal degradation [29]. Autophagy is initiated with activation of the serine/threonine kinase ULK1/2, which forms a complex with ATGs, and the ULK1/2 complex triggers phagophore nucleation by activating the PI3KC3 complex [30, 31]. The LC3 precursor is processed by ATG4, generating LC3-I (soluble LC3 form), which is conjugated to membrane phosphatidylethanolamine (PE) by ATG3, ATG7, and the ATG12-ATG5-ATG16L complex [32]. Conjugated production is membrane-associated lipidated LC3-II that attracts components for phagophore elongation [32]. Phosphoethanolamine cytidylyltransferase (encoded by *PCYT2*) and ethanolamine phosphotransferase 1 (encoded by *SELENOI*) are the two critical enzymes for PE synthesis [33, 34]. In the current study, autophagy, mitophagy, and lysosome pathways were significantly enriched in the medium purple 3 and yellow consensus modules. Relevant genes of the three pathways included *SELENOI*, *PCYT2*, *ULK2*, *BECN1*, *MAP1 LC3A*, *MAP1 LC3B*, *ATG3*, *ATG4B*, *ATG12*, and *ATG9A*, all of which were significantly upregulated in both the FL vs. LL and HG vs. LG. Therefore, we inferred that autophagy initiation might participate in broiler abdominal fat deposition.

## Conclusions

In our current study, we predicted eight consensus modules across four broiler lines (FL and LL, HG and LG), the preservations of which were verified in another broiler line dataset (SRP058295). Pyruvate metabolism, fatty acid metabolism, glycerolipid metabolism, steroid biosynthesis, the PPAR signaling pathway, and the adipocytokine signaling pathway were significantly enriched in the eight consensus modules. Relevant genes of these lipid metabolism pathways included *PDHA1*, *FASN*, *ACSBG2*, *FADS2*, *SCD*, *GPAT3*, *PNPLA7*, *DHCR7*, *PPARG*, *RXRG*, *FABP3*, and *CD36*, all of which were identified in eight consensus modules and significantly upregulated in both the FL vs. LL and HG vs. LG. Based on lipid metabolism pathways enriched in eight consensus modules and overexpression of numerous lipogenic genes, we hypothesize that more fatty acids, TAGs, and cholesterol might be synthesized in broilers with high abdominal fat than in broilers with low abdominal fat. Autophagy, mitophagy, and lysosome were significantly enriched in the eight consensus modules, and the relevant genes of the three pathways, *SELENOI*, *PCYT2*, *ULK2*, *BECN1*, *MAP1 LC3A*, *MAP1 LC3B*, *ATG3*, *ATG4B*, *ATG12*, and *ATG9A,* were significantly upregulated in both FL vs. LL and HG vs. LG. Therefore, we inferred that autophagy initiation might participate in broiler abdominal fat deposition. These genes identified in eight consensus modules can provide targets for further research into abdominal fat deposition in multiple broiler lines. To the best of our knowledge, this is the first report on abdominal fat deposition across multiple different broiler lines via a consensus module analysis method.

## Methods

### RNA-Seq data collection

To reduce the influence of nongenetic factors, such as analytical methods, versions of the genome and annotated files, experimental technologies (RNA-Seq or microarray), sex, diet condition, and weeks of age, we only collected abdominal fat RNA-Seq data of male broilers at 7 weeks of age with normal feed. In the current study, we downloaded GSE42980 [6], GSE49121 [7], and SRP058295 [8] from the GEO (Gene Expression Omnibus) and SRA (Sequence Read Archive) databases of NCBI (National Center for Biotechnology Information).

### Read QC and mapping

To ensure high-quality reads for downstream analyses, quality control (QC) of the RNA-Seq reads was performed using fastp (0.19.7) with default parameters and workflow [35]. After filtering out low-quality reads, the high-quality reads were mapped to the chicken genome using HISAT2 (2.2.0) with default parameters and workflow [36]. The chicken reference genome sequence (*GCF_000002315.6_GRCg6a*) and chicken genome annotation files (*GCF_000002315.6_GRCg6a_genomic.gtf.gz*) were downloaded from the NCBI genome assembly website (ftp://ftp.ncbi.nlm.nih.gov/genomes/all/GCF/000/002/315/GCF_000002315.6_GRCg6a/). Then, the read count of each gene was calculated using featureCounts (v2.0.1) with default parameters [37]. A summary of the RNA-Seq datasets, QC, and mapping is provided in Additional file 1: Table S1.

### Hierarchical cluster analyses of samples

Low-expression genes within the GSE42980 and GSE49121 datasets were filtered using the filterByExpr function in the EdgeR package (3.24.3) [38, 39]. A total of 13,626 genes were retained for subsequent analyses after low expression genes were filtered. The RemoveBatchEffect function in the limma package (3.38.3) was used to remove the batch effect of the GSE42980 dataset [40]. To detect sample clusters within two datasets, we performed hierarchical cluster analyses of samples within GSE42980 and GSE49121, with read counts of 13,626 genes using the hclust function of R software (average method). The hierarchical cluster results of GSE42980 and GSE49121 are shown in Additional file 2: Figure S1.

### Identification of differentially expressed genes

Within DESeq2, read counts from each library were normalized for differences in sequencing depth, used to estimate dispersions from the mean and to fit a linear model according to a negative binomial distribution for each pairwise comparison. Differentially expressed genes (DEGs) within the FL vs. LL (GSE42980) and HG vs. LG (GSE49121) were identified by testing the two factor variables of high- and low-abdominal fat deposition and batch using DEseq2 (1.22.2) with default parameters [41]. The significance threshold for DEGs was set at a false discovery rate adjusted *p*-value (FDR) ≤ 0.05. The significant DEGs in each pairwise comparison are listed in Additional file 3: Table S2.

### Gene coexpression network construction with WGCNA

Gene coexpression networks were constructed using the WGCNA (version 1.68) package in R [42]. The genes were selected for further analyses after passing the quality test by a “goodGene” function in the WGCNA package. The soft threshold (power) was screened to form a scale-free network in both dataset matrices using the “pickSoftThreshold” function in the WGCNA software package. Then, gene coexpression networks for GSE42980 and GSE49121 were separately constructed using the blockwiseModules function in the WGCNA package [10] with these parameters: power = 14, minModuleSize = 30, deepSplit = 2, and mergeCutHeight = 0.3. A consensus gene coexpression network across two datasets was constructed using the blockwiseConsensusModules function in the WGCNA package [10] with these parameters (power = 14, minModuleSize = 30, deepSplit = 2, and mergeCutHeight = 0.3). Consensus modules shared by two gene coexpression networks were detected using the dynamic tree cut method [10, 43].

### Correlation analysis between consensus modules and abdominal fat deposition

The module eigengene is defined as the first principal component of a gene module [10]. To identify modules of interest, the correlation between each module eigengene of consensus modules and abdominal fat deposition traits was calculated using the correlation analysis function in the WGCNA package [10], and significant consensus modules were identified with the threshold (*p*-value ≤0.05).

### Preservation test of consensus modules

To test the preservation of consensus modules significantly correlated with abdominal fat deposition in other broiler lines, abdominal fat RNA-Seq data of male broilers (SRP058295) were downloaded to construct a test network for preservation analysis. The preservation analysis of consensus modules was performed using the modulePreservation function in the WGCNA package with the parameters nPermutations = 200, randomSeed = 1, quickCor = 0, verbose = 3 [11]. The *medianRank* was used to compare relative preservation among consensus modules, and a module with higher median rank exhibits weaker observed preservation statistics than a module with a lower median rank [11]. The thresholds of *Zsummary* were set as follows: *Zsummary* > 10 means strong evidence of preservation; 2 < *Zsummary* < 10 means weak to moderate evidence of preservation; *Zsummary* < 2 means no evidence of preservation [11].

### Function enrichment of consensus modules

Based on KEGG (Kyoto Encyclopedia of Genes and Genomes) and GO (Gene Ontology) databases, the pathways and GO terms associated with the genes within significant consensus modules were predicted using the clusterprofiler software package (3.10.1) [44]. The significance threshold for the pathways and GO terms associated with the consensus modules was set at a *p*-value ≤0.05.

## Supplementary Information

**Additional file 1: Table S1.** Summary of RNA-Seq data, QC, and read mapping.

**Additional file 2: Figure S1.** Hierarchical cluster of samples within GSE42980 and GSE49121.

**Additional file 3: Table S2.** Summary of genes within consensus modules and DEGs within FL vs. LL and HG vs. LG.

**Additional file 4: Figure S2.** Cluster dendrograms of consensus, GSE42980, and GSE49121 modules.

**Additional file 5: Figure S3.** Heatmaps of consensus modules correlated with abdominal fat deposition within GSE42980 or GSE49121.

**Additional file 6: Table S3.** Functional enrichment correlated with genes within eight consensus modules.

## Data Availability

Publicly available datasets were used for analysis in the current study, and the datasets consisted of GSE42980, GSE49121 and SRP058295. GSE42980 and GSE49121 are available in NCBI’s Gene Expression Omnibus (https://www.ncbi.nlm.nih.gov/gds/?term=GSE42980;https://www.ncbi.nlm.nih.gov/gds/?term=GSE49121), and SRP058295 is available in NCBI’s SRA database (https://www.ncbi.nlm.nih.gov/sra/?term=SRP058295). The chicken reference genome sequence (*GCF_000002315.6_GRCg6a*) and chicken genome annotation files (*GCF_000002315.6_GRCg6a_genomic.gtf.gz*) were downloaded from the NCBI genome assembly website (ftp://ftp.ncbi.nlm.nih.gov/genomes/all/GCF/000/002/315/GCF_000002315.6_GRCg6a/).

## Abbreviations

DEGs: Differentially expressed genes
RNA-Seq: RNA-sequencing
WGCNA: Weighted gene co-expression network analysis
FL: Fat line
LL: Lean line
HG: High growth
LG: Low growth
PDHA1: Pyruvate dehydrogenase (lipoamide) alpha 1
FASN: Fatty acid synthase
ACSBG2: Acyl-CoA synthetase bubblegum family member 2
FADS2: Fatty acid desaturase 2
SCD: Stearoyl-CoA desaturase
DHCR7: 7-dehydrocholesterol reductase
PPARG: Peroxisome proliferator-activated receptor gamma
RXRG: Retinoid X receptor gamma
FABP3: Fatty acid binding protein 3
CD36: CD36 molecule
TAGs: Triacylglycerols
AFP: Abdominal fat percentage
HFE: High feed efficiency
LFE: Low feed efficiency
NEAU: Northeast Agricultural University
KEGG: Kyoto Encyclopedia of Genes and Genomes
GO: Gene Ontology
DLAT: Dihydrolipoamide S-acetyltransferase
ACOX1: Acyl-CoA oxidase 1
DGAT2: Diacylglycerol O-acyltransferase 2
ACSL4: Acyl-CoA synthetase long chain family member 4
SUCLA2: Succinate-CoA ligase ADP-forming beta subunit
ACAT2: Acetyl-CoA acetyltransferase 2
GPAT3: Glycerol-3-phosphate acyltransferase 3
GPD2: Glycerol-3-phosphate dehydrogenase 2
PNPLA7: Patatin like phospholipase domain containing 7
ETNPPL: Ethanolamine-phosphate phospho-lyase
GNAI3: G protein subunit alpha i3
ACADS: Acyl-CoA dehydrogenase, C-2 to C-3 short chain
PPT1: Palmitoyl-protein thioesterase 1
HACD1: 3-hydroxyacyl-CoA dehydratase 1
HACD2: 3-hydroxyacyl-CoA dehydratase 2
MCAT: Malonyl-CoA-acyl carrier protein transacylase
ULK2: Unc-51 like autophagy activating kinase 2
ATG3: Autophagy related 3
ATG12: Autophagy related 12
ATG9A: Autophagy related 9A
ATG4B: Autophagy related 4B cysteine peptidase
MAP1LC3A: Microtubule associated protein 1 light chain 3 alpha
MAP1LC3B: Microtubule associated protein 1 light chain 3 beta
BECN1: Beclin 1
PCYT2: Phosphate cytidylyltransferase 2, ethanolamine
SELENOI: Selenoprotein I
SC5D: Sterol-C5-desaturase
VLDL: Very low density lipoprotein
ApoE: Apolipoprotein E
GEO: Gene Expression Omnibus
SRA: Sequence Read Archive
NCBI: National Center for Biotechnology Information
QC: Quality control
FDR: False discovery rate

## References

[1] Daniel CR, Cross AJ, Koebnick C, Sinha R (2011). Trends in meat consumption in the USA. Public Health Nutr.

[2] Sokoya OO, Babajide JM, Shittu TA, Sanwo KA, Adegbite JA (2019). Chemical and color characterization of breast meat from FUNAAB indigenous and marshal broiler chickens. Trop Anim Health Pro.

[3] Baeza E, Le Bihan-Duval E (2013). Chicken lines divergent for low or high abdominal fat deposition: a relevant model to study the regulation of energy metabolism. Animal..

[4] Abdalla BA, Chen J, Nie Q, Zhang X (2018). Genomic insights into the multiple factors controlling abdominal fat deposition in a chicken model. Front Genet.

[5] Wang G, Kim WK, Cline MA, Gilbert ER (2017). Factors affecting adipose tissue development in chickens: a review. Poultry Sci.

[6] Resnyk CW, Chen C, Huang H, Wu CH, Simon J, Le Bihan-Duval E, Duclos MJ, Cogburn LA (2015). RNA-Seq analysis of abdominal fat in genetically fat and lean chickens highlights a divergence in expression of genes controlling adiposity, hemostasis, and lipid metabolism. PLoS One.

[7] Resnyk CW, Carre W, Wang X, Porter TE, Simon J, Le Bihan-Duval E, Duclos MJ, Aggrey SE, Cogburn LA (2017). Transcriptional analysis of abdominal fat in chickens divergently selected on bodyweight at two ages reveals novel mechanisms controlling adiposity: validating visceral adipose tissue as a dynamic endocrine and metabolic organ. BMC Genomics.

[8] Zhuo Z, Lamont SJ, Lee WR, Abasht B (2015). RNA-Seq analysis of abdominal fat reveals differences between modern commercial broiler chickens with high and low feed efficiencies. PLoS One.

[9] Wang HB, Li H, Wang QG, Zhang XY, Wang SZ, Wang YX, Wang XP (2007). Profiling of chicken adipose tissue gene expression by genome array. BMC Genomics.

[10] Langfelder P, Horvath S (2007). Eigengene networks for studying the relationships between co-expression modules. BMC Syst Biol.

[11] Langfelder P, Luo R, Oldham MC, Horvath S (2011). Is my network module preserved and reproducible?. PLoS Comput Biol.

[12] Resnyk CW, Carre W, Wang X, Porter TE, Simon J, Le Bihan-Duval E, Duclos MJ, Aggrey SE, Cogburn LA (2013). Transcriptional analysis of abdominal fat in genetically fat and lean chickens reveals adipokines, lipogenic genes and a link between hemostasis and leanness. BMC Genomics.

[13] Clarke SD (1993). Regulation of fatty acid synthase gene expression: an approach for reducing fat accumulation. J Anim Sci.

[14] Ma X, Sun J, Zhu S, Du Z, Li D, Li W, Li Z, Tian Y, Kang X, Sun G (2020). MiRNAs and mRNAs Analysis during Abdominal Preadipocyte Differentiation in Chickens. Animals.

[15] Zhang M, Li F, Ma XF, Li WT, Jiang RR, Han RL, Li GX, Wang YB, Li ZY, Tian YD (2019). Identification of differentially expressed genes and pathways between intramuscular and abdominal fat-derived preadipocyte differentiation of chickens in vitro. BMC Genomics.

[16] Cui X, Cui H, Liu L, Zhao G, Liu R, Li Q, Zheng M, Wen J (2018). Decreased testosterone levels after caponization leads to abdominal fat deposition in chickens. BMC Genomics.

[17] Chang P, Sun T, Heier C, Gao H, Xu H, Huang F (2020). Interaction of the Lysophospholipase PNPLA7 with lipid droplets through the catalytic region. Mol Cells.

[18] Wang X, Guo M, Wang Q, Wang Q, Zuo S, Zhang X, Tong H, Chen J, Wang H, Chen X (2020). The Patatin-like phospholipase domain containing protein 7 facilitates VLDL secretion by modulating ApoE stability. Hepatology..

[19] Cao J, Li JL, Li D, Tobin JF, Gimeno RE (2006). Molecular identification of microsomal acyl-CoA:glycerol-3-phosphate acyltransferase, a key enzyme in de novo triacylglycerol synthesis. P Natl Acad Sci USA.

[20] Chitraju C, Walther TC, Farese RV (2019). The triglyceride synthesis enzymes DGAT1 and DGAT2 have distinct and overlapping functions in adipocytes. J Lipid Res.

[21] Jiang XS, Backlund PS, Wassif CA, Yergey AL, Porter FD (2010). Quantitative proteomics analysis of inborn errors of cholesterol synthesis: identification of altered metabolic pathways in DHCR7 and SC5D deficiency. Mol Cell Proteomics.

[22] Cui TT, Xing TY, Chu YK, Li H, Wang N (2017). Genetic and epigenetic regulation of PPARgamma during adipogenesis. Yi chuan.

[23] Sato K, Abe H, Kono T, Yamazaki M, Nakashima K, Kamada T, Akiba Y (2009). Changes in peroxisome proliferator-activated receptor gamma gene expression of chicken abdominal adipose tissue with different age, sex and genotype. Anim Sci J.

[24] Wang Y, Mu Y, Li H, Ding N, Wang Q, Wang Y, Wang S, Wang N (2008). Peroxisome proliferator-activated receptor-gamma gene: a key regulator of adipocyte differentiation in chickens. Poultry Sci..

[25] Cheng S, Wang M, Wang Y, Zhang C, Wang Y, Song J, Zuo Q, Zhang Y, Li B (2018). RXRG associated in PPAR signal regulated the differentiation of primordial germ cell. J Cell Biochem.

[26] Shu G, Liao WY, Feng JY, Yu KF, Zhai YF, Wang SB, Khondowe P, Wang XQ, Jiang QY (2011). Active immunization of fatty acid translocase specifically decreased visceral fat deposition in male broilers. Poultry Sci..

[27] Mizushima N, Levine B (2010). Autophagy in mammalian development and differentiation. Nat Cell Biol.

[28] Romero M, Zorzano A (2019). Role of autophagy in the regulation of adipose tissue biology. Cell Cycle.

[29] Ferhat M, Funai K, Boudina S (2019). Autophagy in adipose tissue physiology and pathophysiology. Antioxid Redox Sign.

[30] Jung CH, Jun CB, Ro SH, Kim YM, Otto NM, Cao J, Kundu M, Kim DH (2009). ULK-Atg13-FIP200 complexes mediate mTOR signaling to the autophagy machinery. Mol Biol Cell.

[31] Chang YY, Neufeld TP (2009). An Atg1/Atg13 complex with multiple roles in TOR-mediated autophagy regulation. Mol Biol Cell.

[32] Clemente-Postigo M, Tinahones A, El Bekay R, Malagon MM, Tinahones FJ (2020). The Role of Autophagy in White Adipose Tissue Function: Implications for Metabolic Health. Metabolites.

[33] Vaz FM, McDermott JH, Alders M, Wortmann SB, Kolker S, Pras-Raves ML, Vervaart MAT, van Lenthe H, Luyf ACM, Elfrink HL (2019). Mutations in PCYT2 disrupt etherlipid biosynthesis and cause a complex hereditary spastic paraplegia. Brain..

[34] Ahmed MY, Al-Khayat A, Al-Murshedi F, Al-Futaisi A, Chioza BA, Pedro Fernandez-Murray J, Self JE, Salter CG, Harlalka GV, Rawlins LE (2017). A mutation of EPT1 (SELENOI) underlies a new disorder of Kennedy pathway phospholipid biosynthesis. Brain..

[35] Chen S, Zhou Y, Chen Y, Gu J (2018). Fastp: an ultra-fast all-in-one FASTQ preprocessor. Bioinformatics..

[36] Kim D, Langmead B, Salzberg SL (2015). HISAT: a fast spliced aligner with low memory requirements. Nat Methods.

[37] Liao Y, Smyth GK, Shi W (2014). featureCounts: an efficient general purpose program for assigning sequence reads to genomic features. Bioinformatics..

[38] Robinson MD, McCarthy DJ, Smyth GK (2010). edgeR: a Bioconductor package for differential expression analysis of digital gene expression data. Bioinformatics..

[39] McCarthy DJ, Chen Y, Smyth GK (2012). Differential expression analysis of multifactor RNA-Seq experiments with respect to biological variation. Nucleic Acids Res.

[40] Ritchie ME, Phipson B, Wu D, Hu Y, Law CW, Shi W, Smyth GK (2015). limma powers differential expression analyses for RNA-sequencing and microarray studies. Nucleic Acids Res.

[41] Love MI, Huber W, Anders S (2014). Moderated estimation of fold change and dispersion for RNA-seq data with DESeq2. Genome Biol.

[42] Langfelder P, Horvath S (2008). WGCNA: an R package for weighted correlation network analysis. BMC Bioinformatics.

[43] Langfelder P, Zhang B, Horvath S (2008). Defining clusters from a hierarchical cluster tree: the dynamic tree cut package for R. Bioinformatics..

[44] Yu G, Wang LG, Han Y, He QY (2012). clusterProfiler: an R package for comparing biological themes among gene clusters. Omics..

