# Supplementary figures and images for: Consensus module analysis of abdominal fat deposition across multiple broiler lines
Source: BMC Genomics. 2021 Feb 10;22:115. doi: 10.1186/s12864-021-07423-6 (PMC7876793; doi:10.1186/s12864-021-07423-6)

**A****Sample clustering on all genes in GSE42980**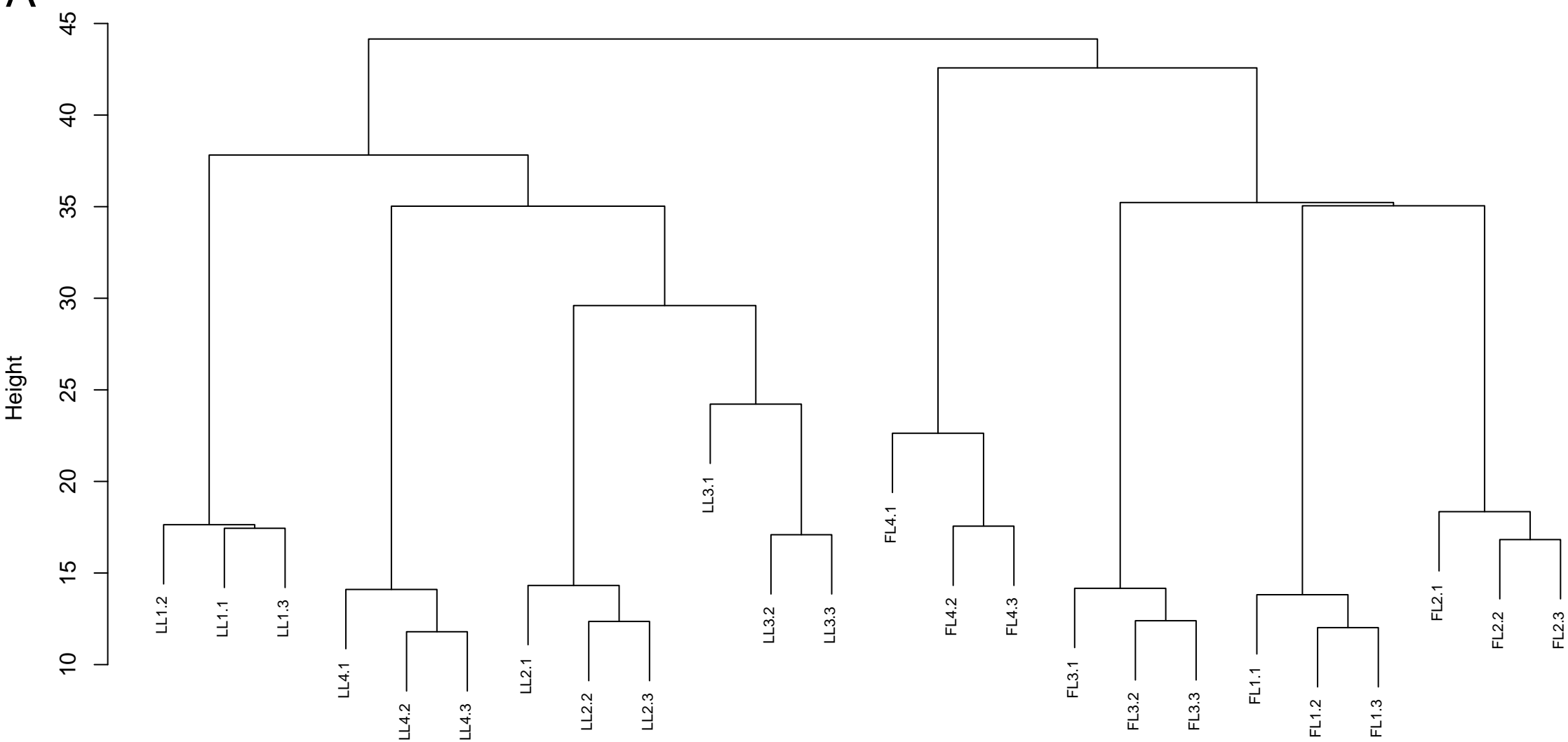**B****Sample clustering on all genes in GSE49121**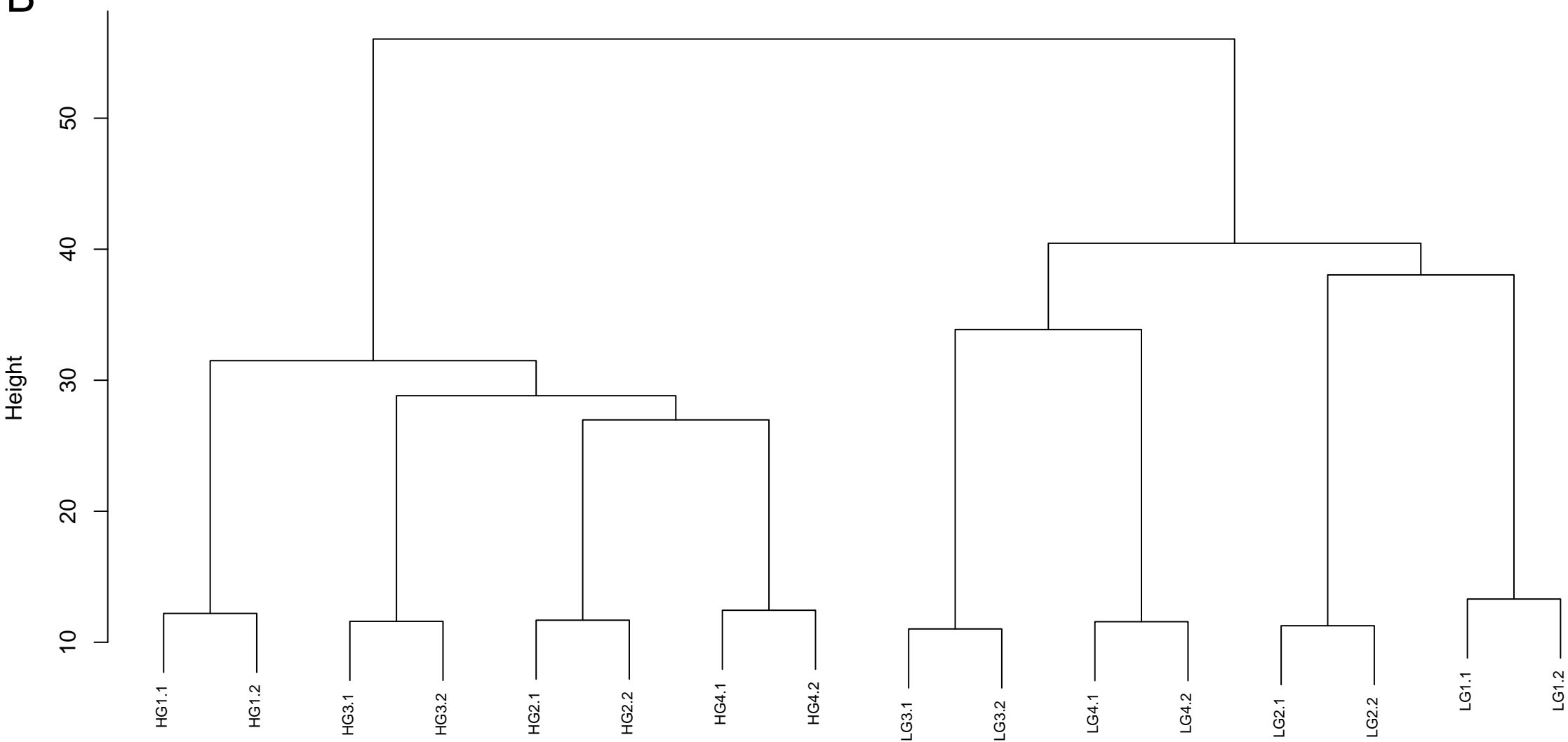

Supplement: Supplementary file 2 — Additional file 2: Figure S1. Hierarchical cluster of samples within GSE42980 and GSE49121. [file 12864_2021_7423_MOESM2_ESM.pdf]

A

Module--trait relationships in GSE42980

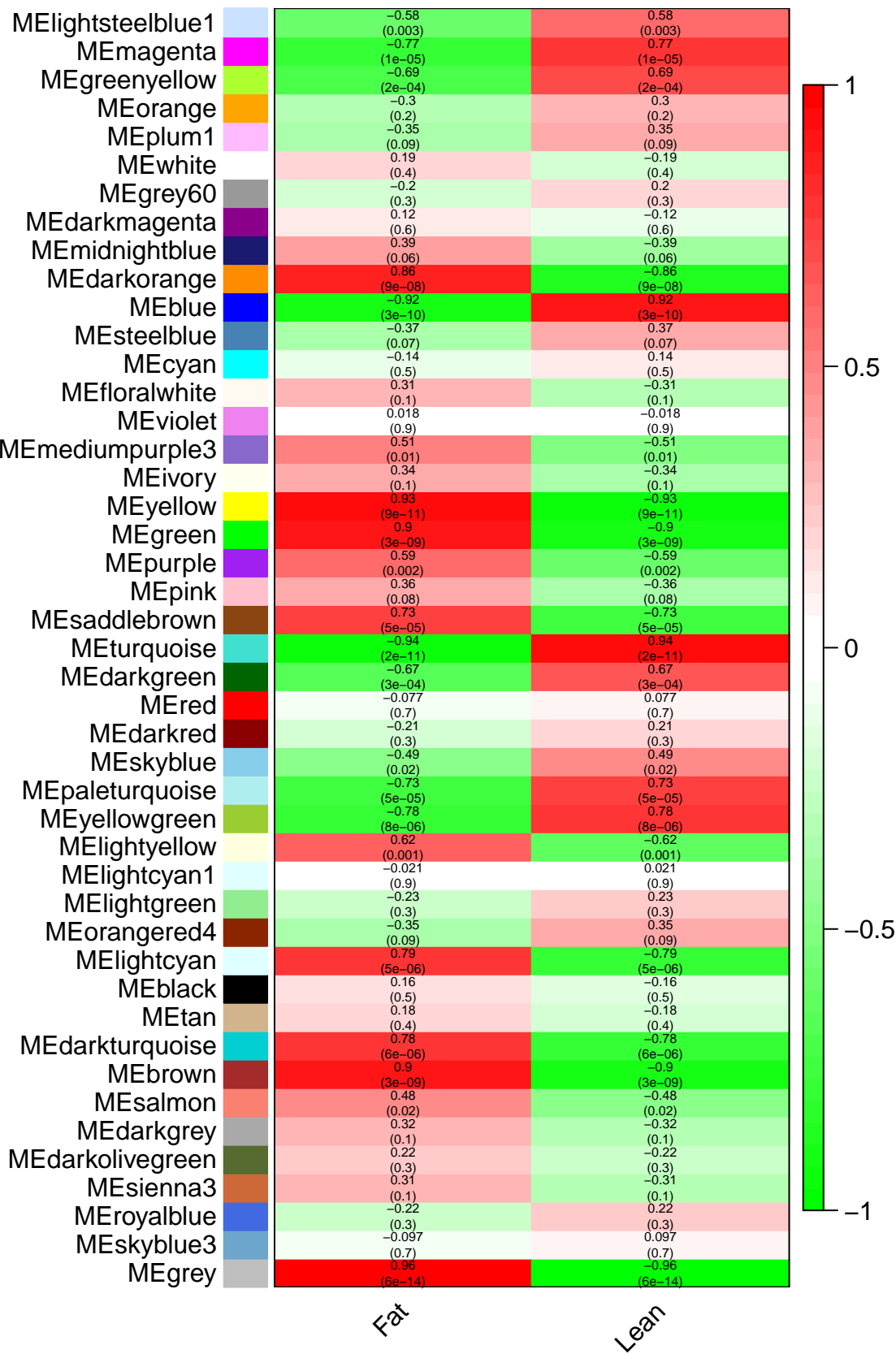

B

Module--trait relationships in GSE49121

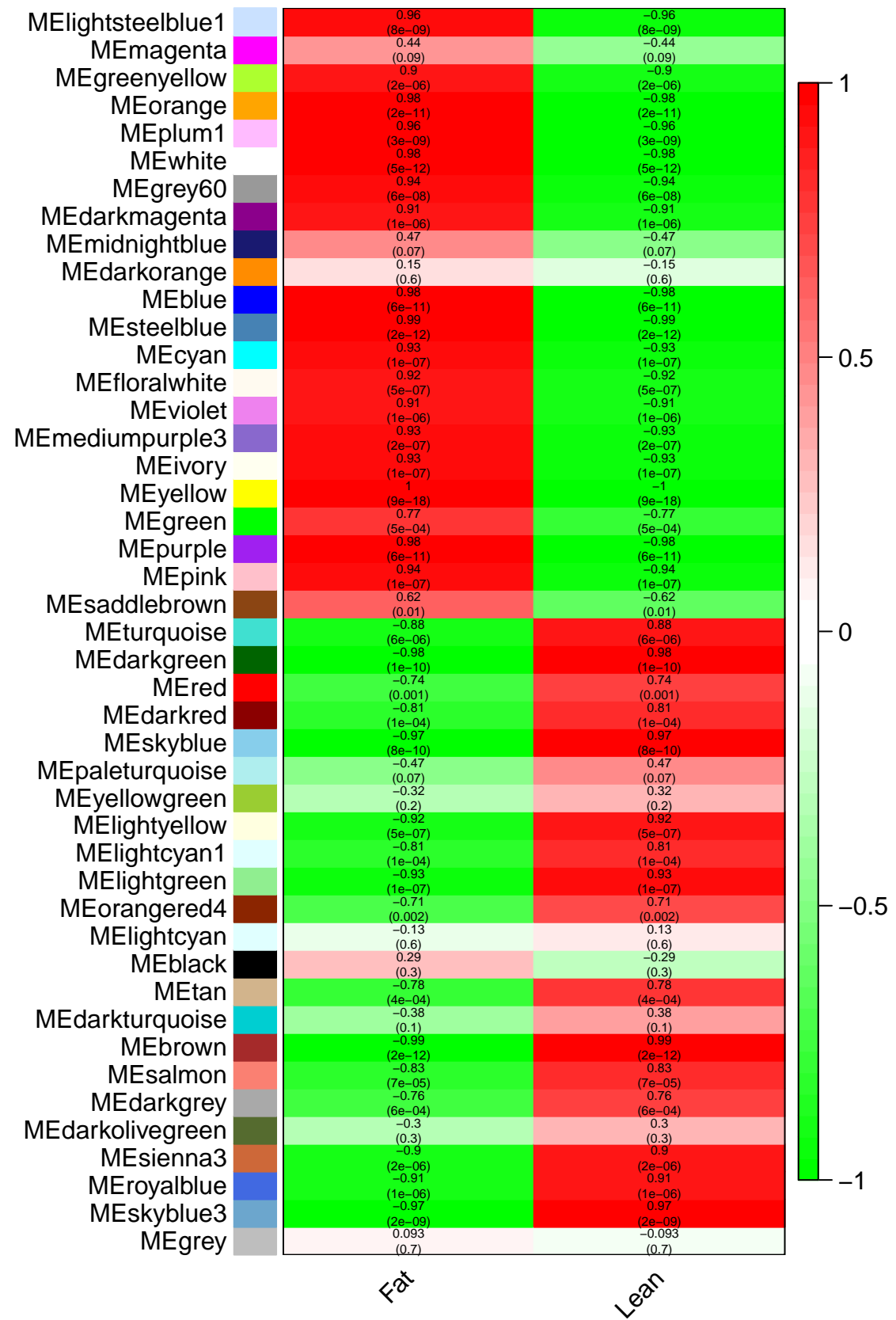

Supplement: Supplementary file 5 — Additional file 5: Figure S3. Heatmaps of consensus modules correlated with abdominal fat deposition within GSE42980 or GSE49121. [file 12864_2021_7423_MOESM5_ESM.pdf]
