# Supplementary material for: Consensus module analysis of abdominal fat deposition across multiple broiler lines
Source: BMC Genomics. 2021 Feb 10;22:115. doi: 10.1186/s12864-021-07423-6 (PMC7876793; doi:10.1186/s12864-021-07423-6)
Supplement: Supplementary file 4 — Additional file 4: Figure S2. Cluster dendrograms of consensus, GSE42980, and GSE49121 modules. [file 12864_2021_7423_MOESM4_ESM.pdf]

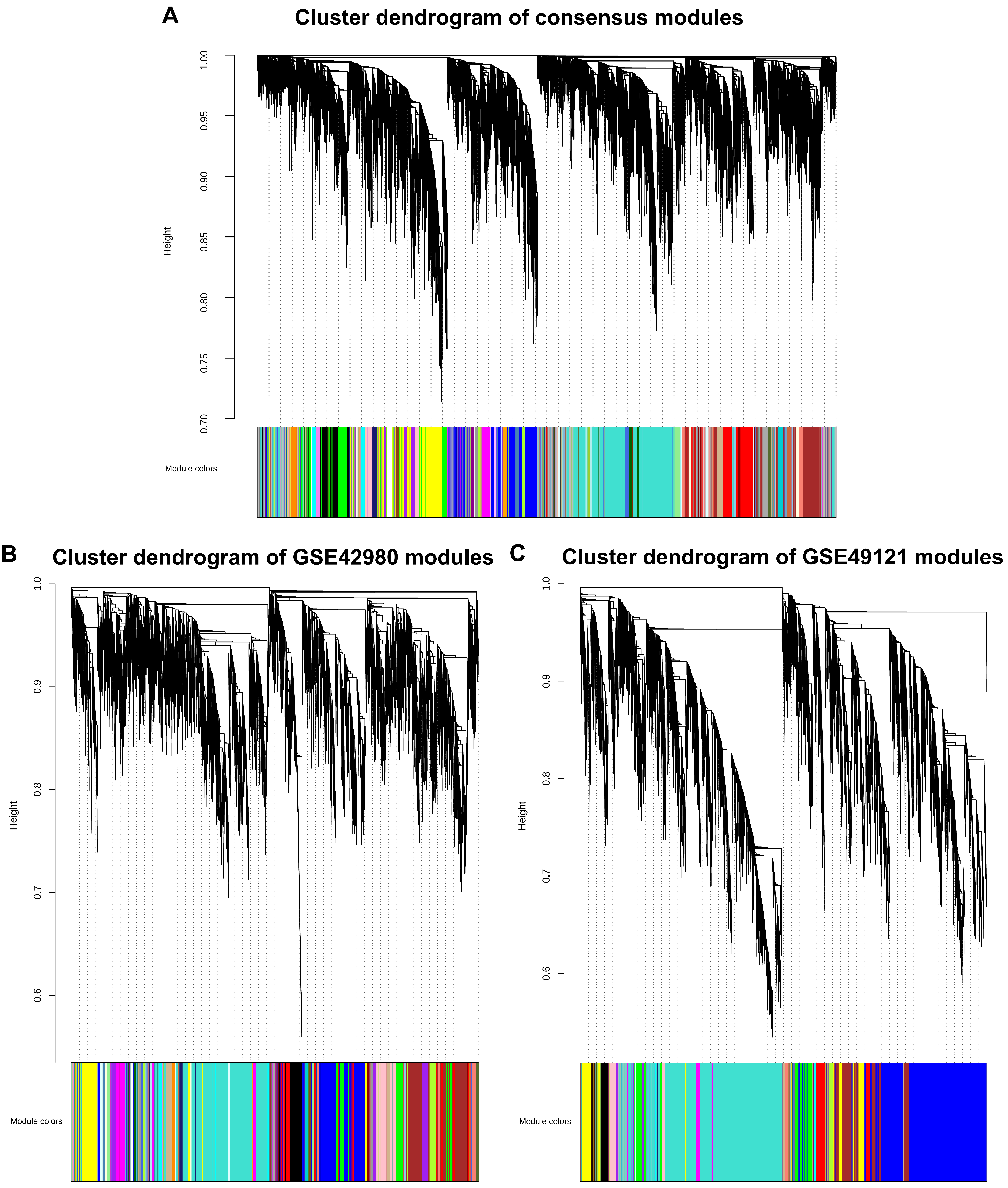
A
Cluster dendrogram of consensus modules
Height
1.00
0.95
0.90
0.85
0.80
0.75
0.70
Module colors
B
Cluster dendrogram of GSE42980 modules
Height
1.0
0.9
0.8
0.7
0.6
Module colors
C
Cluster dendrogram of GSE49121 modules
Height
1.0
0.9
0.8
0.7
0.6
Module colors
